# Risk factors of neurologic deficit after thoracolumbar burst fracture

**DOI:** 10.3389/fneur.2025.1542622

**Published:** 2025-05-12

**Authors:** Yuxi Liu, Shilin Zhang, Daxiong Feng, Jing Luo, Hong Zhang, Likun Wang

**Affiliations:** ^1^Department of Orthopaedics, Santai People’s Hospital, Mianyang, China; ^2^Department of Orthopaedics, The Sixth People’s Hospital of Chengdu, Chengdu, China; ^3^Department of Orthopaedics, The Affiliated Hospital of Southwest Medical University, Luzhou, China; ^4^Department of Rehabilitation, The Third Hospital of Mianyang, Sichuan Mental Health Center, Mianyang, China

**Keywords:** thoracolumbar, burst fracture, neurologic deficit, spinal fractures, risk factors

## Abstract

**Introduction:**

Traumatic fractures of the vertebral bodies in the thoracolumbar region are the most common type of spinal fractures. Some studies suggest that neurological deficits associated with these fractures may be linked to spinal canal compromise, kyphosis angle, and other factors. However, this relationship remains controversial. The present study aimed to identify the risk factors for neurologic deficits following thoracolumbar burst fractures (TBF).

**Methods:**

This study included 322 patients with TBF, comprising 115 patients with lamina fractures (LF) and 207 patients without lamina fractures (NLF). Neurological deficits were classified according to the American Spinal Injury Association (ASIA) classification, with 75 patients exhibiting neurological deficits and 247 patients without deficits. Clinical data, ASIA classification, and imaging findings were analyzed and compared between the two groups. Risk factors were assessed using logistic regression.

**Results:**

Compared with the NLF group, the LF group had higher ASIA classification scores (*P* < 0.05). Multivariate logistic regression identified laminar fracture (OR: 0.019, 95% CI: 0.005–0.070, *P* < 0.000), car accident (OR: 6.082, 95% CI: 1.248–29.636, *P* = 0.025), and falling accident (OR: 8.429, 95% CI: 2.143–33.153, *P* = 0.002) as independent variables associated with neurologic deficit. Additionally, the ROC curve revealed that laminar fractures and falling accidents had a high risk association value. A risk association equation, Logit (P) = −4.358 + 3.535 × laminar fracture – 1.353 × falling accidents, was established based on the high-value indicators.

**Conclusion:**

Laminar fractures, car accidents, and falls were identified as independent risk factors for neurological deficits following TBF. Additionally, laminar fractures and falls demonstrated a high risk association value. These findings provide valuable insights for optimizing rehabilitation strategies and guiding surgical decision-making.

## Introduction

Studies show that traumatic burst fractures of the thoracolumbar spine are the most prevalent type of spinal injury (1). These fractures involve the anterior and middle columns of the spine, with or without involvement of the posterior column (2). However, they can also result in disruption of the vertebral lamina and lead to spinal cord injury.

Thoracolumbar burst fractures (TBF) usually result from axial load mechanisms, which concentrate pressure on the medial column of the spine, leading to a widening of the gap between the pedicles and potentially causing a vertical laminar fracture. Laminar fractures, also known as “greenstick fractures,” typically occur in the ventral cortex of the lamina (3). Reports have shown a correlation between laminar fractures and dural tears (4). However, the role of laminar fractures in neurologic deficits is often underestimated, as the course of the injury is dynamic, and the lamina plays a crucial role in assessing stability across the fracture segment.

There are fewer relevant studies focusing on the risk factors for neurological deficits after TBF. In this study, we analyzed and compared patients’ clinical data, neurological deficit classification, and imaging findings, and assessed the risk factors using logistic regression. A better understanding of the risk-associated factors for neurological deficits following TBF enables clinicians to provide more accurate information to patients and their families and to develop more tailored treatment and rehabilitation programs based on expected outcomes.

## Materials and methods

This study adhered to the principles outlined in the Helsinki Declaration and received approval from our institution’s medical ethics committee. Due to the retrospective nature of the study, the ethics committee waived the requirement for informed consent. We conducted a retrospective analysis of 322 patients with TBF who were admitted to our institution between November 2012 and November 2022. Among them, 115 had laminar fractures (LF), and 207 had no laminar fractures (NLF). Neurological deficits were classified according to the American Spinal Injury Association (ASIA) classification upon patient admission to the hospital (5). Patients with neurological deficits (ASIA A, ASIA B, ASIA C, and ASIA D) were grouped together and compared with those without neurological deficits (ASIA E), comprising 75 and 247 patients, respectively. The inclusion criteria were as follows: (1) TBF diagnosed according to Denis’ classification system (2); (2) a clear history of trauma; (3) fracture segment located between T11 and L2; and (4) clearly documented ASIA grading. The exclusion criteria included: (1) multiple contiguous or non-contiguous spinal fractures; (2) pathological or old fractures; (3) pre-existing neurological deficits; (4) penetrating injuries; and (5) incomplete clinical data.

After screening the cases that met the inclusion criteria, we collected and compared each patient’s age, gender, smoking status, alcohol consumption, history of hypertension and diabetes, disease duration, mechanism of trauma, fractured vertebral segment, ISSM score, ASIA classification, and presence of laminar fracture. The injury mechanisms included blows from falling objects, car accidents, falls, and other causes. Trauma severity was assessed using the Injury Severity Score (ISS) (6), which was determined based on the Abbreviated Injury Scale-2005 (7). In this study, to investigate the relationship between laminar fractures and neurological deficits, components related to neurological deficits and laminar fractures were excluded from the ISS calculation, resulting in a modified score, the ISSM.

### Statistical analysis

Data were analyzed using the Statistical Package for the Social Sciences (SPSS) software version 23 and visualized with GraphPad Prism 9 software. Continuous variables were expressed as mean ± standard deviation (SD), and between-group comparisons were performed using independent t-tests. Categorical variables were presented as counts and percentages, with comparisons assessed using the chi-square test. Ordinal data were analyzed using the Kruskal-Wallis test. Binary logistic regression was conducted to identify risk factors. The Hosmer-Lemeshow test was used to evaluate the model’s goodness-of-fit, and the receiver operating characteristic (ROC) curve was analyzed to assess discrimination. All statistical tests were two-tailed, and a *P*-value < 0.05 was considered statistically significant.

## Results

### Clinical data and ASIA classification comparison

Clinical data, including age, gender, smoking status, alcohol consumption, hypertension, diabetes, disease course, mechanism of trauma, fractured vertebral segment, and ISSM score, showed no statistically significant differences between the LF and NLF groups (*P* > 0.05, Table 1). However, the ASIA classification score in the LF group was significantly higher than in the NLF group (*P* < 0.05, Table 2). In the LF group, 5 patients (4.3%) were classified as ASIA A, 6 patients (5.2%) as ASIA B, 16 patients (13.9%) as ASIA C, 33 patients (28.7%) as ASIA D, and 55 patients (47.8%) as ASIA E. In contrast, in the NLF group, no patients (0.0%) were classified as ASIA A, 2 patients (1.0%) as ASIA B, 1 patient (0.5%) as ASIA C, 12 patients (5.8%) as ASIA D, and 192 patients (92.8%) as ASIA E.

**Table 1:** Clinical data.

Factor	No Lamina fracture group (*n* = 207)	Lamina fracture group (*n* = 115)	*χ^2^*-value	*P*-value
Age≥40 years (*n* = 245)	153 (73.9)	92 (80.0)	1.505	0.220
Male (*n* = 227)	143 (69.1)	84 (73.0)	0.558	0.455
Smoking habit (*n* = 53)	34 (16.4)	19 (16.5)	0.001	0.982
Alcohol consumption (*n* = 44)	25 (12.1)	19 (16.5)	1.238	0.266
Hypertension (*n* = 24)	16 (7.7)	8 (7.0)	0.064	0.800
Diabetes (*n* = 5)	2 (1.0)	3 (2.6)	0.451	0.502
Course of disease≥3 d (*n* = 63)	45 (21.7)	18 (15.7)	1.741	0.187
Mechanism of trauma				
Car (*n* = 44)	30 (14.5)	14 (12.2)	5.134	0.162
Fall (*n* = 190)	113 (54.6)	77 (67.0)		
Blow (*n* = 19)	13 (6.3)	6 (5.2)		
Other (*n* = 69)	51 (24.6)	18 (15.7)		
Fractured vertebral segment				
T11 (*n* = 8)	5 (2.4)	3 (2.6)	0.849	0.838
T12 (*n* = 53)	36 (17.4)	17 (14.8)		
L1 (*n* = 166)	103 (49.8)	63 (54.8)		
L2 (*n* = 95)	63 (30.4)	32 (27.8)		
ISSM				
≤16 (*n* = 259)	169 (81.6)	90 (78.3)	0.659	0.417
>16 (*n* = 59)	37 (17.9)	22 (19.1)		
>25 (*n* = 4)	1 (0.5)	3 (2.6)		

T, Thoracic; L, Lumbar; ISSM, Injury Severity Score with modification. *P-values derived from chi-square test.

**Table 2:** ASIA classification.

Factor	No Lamina fracture group (*n* = 207)	Lamina fracture group (*n* = 115)	*χ^2^*-value	*P*-value
A (*n* = 5)	0 (0.0)	5 (4.3)	85.080	<0.000
B (*n* = 8)	2 (1.0)	6 (5.2)		
C (*n* = 17)	1 (0.5)	16 (13.9)		
D (*n* = 45)	12 (5.8)	33 (28.7)		
E (*n* = 247)	192 (92.8)	55 (47.8)		

A, Sacral segment losses motor or sensory function; B, Sensory function remains below the nerve plane (such as sacral segment S4–S5), without motor function; C, Motor function exists below the plane (most key muscle strength <grade 3); D, Motor function remains as well (strength >grade 3); E, Sensory and motor functions were normal. *P-values derived from Kruskal-Wails test.

### Risk factors of neurologic deficit after TBF

**Figure 1:**
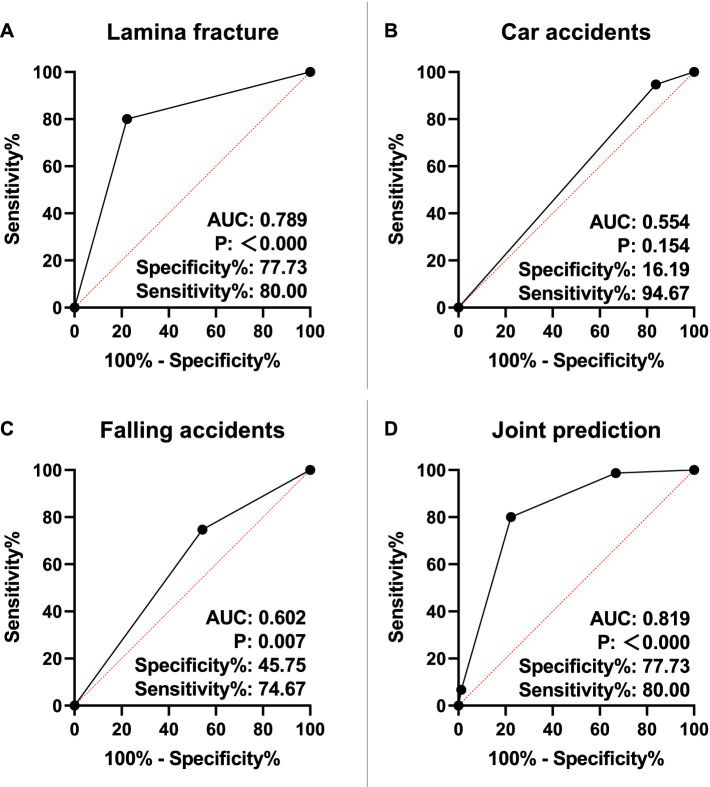
Value of lamina fracture, car and falling accidents in neurologic deficit. **(A)** Risk association value of lamina fracture in neurologic deficit. **(B)** Risk association value of car accidents in neurologic deficit. **(C)** Risk association value of falling accidents in neurologic deficit. **(D)** ROC curve of neurologic deficit associated by risk association equation. AUC, area under the curve.

Univariate analysis revealed that laminar fractures and the mechanism of trauma (car accidents, falls, and other accidents) were associated with neurological deficits following TBF (*P* < 0.05, Table 3). Subsequently, variables showing significant differences were included in the logistic regression analysis and tested using the backward LR method. Laminar fractures (OR: 0.019, 95% CI: 0.005–0.070), car accidents (OR: 6.082, 95% CI: 1.248–29.636), and falls (OR: 8.429, 95% CI: 2.143–33.153) were independently associated with neurological deficits after TBF (*P* < 0.05, Table 4). Furthermore, ROC curves were plotted to assess the risk association value of each index for neurological deficits following TBF. The results indicated that laminar fractures and falls had a high risk association value (*P* < 0.01, Figure 1). Finally, the risk association equation Logit (P) = −4.358 + 3.535 × laminar fracture – 1.353 × falling accidents was established based on high-value indicators, and the goodness-of-fit was assessed using the Hosmer-Lemeshow test (*P* = 0.324). The area under the curve (AUC) for associating neurological deficits in TBF patients was 0.819 (95% CI: 0.769–0.869, *P* < 0.000), indicating good model performance.

**Table 3:** Univariate analysis.

Variable	No Neurologic deficit group (*n* = 247)	Neurologic deficit group (*n* = 75)	OR (95%Cl)	*P*-value
Age≥40 years (*n* = 245)	191 (77.3)	54 (72.0)	1.326 (0.739–2.382)	0.344
Male (*n* = 227)	171 (69.2)	56 (74.7)	0.763 (0.425–1.372)	0.367
Smoking habit (*n* = 53)	39 (15.8)	14 (18.7)	0.817 (0.416–1.603)	0.557
Alcohol consumption (*n* = 44)	30 (12.1)	14 (18.7)	0.602 (0.301–1.207)	0.153
Hypertension (*n* = 24)	21 (8.5)	3 (4.0)	2.230 (0.646–7.694)	0.204
Diabetes (*n* = 5)	3 (1.2)	2 (2.7)	0.449 (0.074–2.737)	0.385
Course of disease≥3 d (*n* = 63)	52 (21.1)	11 (14.7)	1.552 (0.763–3.153)	0.225
Lamina fracture (*n* = 115)	55 (22.3)	60 (80.0)	0.072 (0.038–0.136)	<0.000
Mechanism of trauma				0.005
Car (*n* = 44)	40 (16.2)	4 (5.3)	3.430 (1.185–9.926)	0.023
Fall (*n* = 190)	134 (54.3)	56 (74.7)	0.402 (0.226–0.717)	0.002
Blow (*n* = 19)	13 (5.3)	6 (8.0)	0.639 (0.234–1.743)	0.382
Other (*n* = 69)	60 (24.3)	9 (12.0)	2.353 (1.106–5.005)	0.026
Fractured vertebral segment				0.541
T11 (*n* = 8)	6 (2.4)	2 (2.7)	0.909 (0.180–4.599)	0.908
T12 (*n* = 53)	42 (17.0)	11 (14.7)	1.192 (0.580–2.451)	0.633
L1 (*n* = 166)	122 (49.4)	44 (58.7)	0.688 (0.408–1.160)	0.160
L2 (*n* = 95)	77 (31.2)	18 (24.0)	1.434 (0.792–2.599)	0.234
ISSM				0.306
≤16 (*n* = 259)	201 (81.4)	58 (77.3)	1.281 (0.683–2.401)	0.440
>16 (*n* = 59)	44 (17.8)	15 (20.0)	0.867 (0.451–1.666)	0.668
>25 (*n* = 4)	2 (0.8)	2 (2.7)	0.298 (0.041–2.152)	0.230

T, Thoracic; L, Lumbar; ISSM, Injury Severity Score with modification; OR, odds ratio; Cl, confidence interval. *P-values derived from binary logistic regression analysis.

**Table 4:** Logistics regression.

Variable	*β*	SE	Wals	Sig	Exp(B)	95%Cl
Lamina fracture	−3.940	0.654	36.289	<0.000	0.019	0.005–0.070
Mechanism of trauma						
Car	1.805	0.808	4.993	0.025	6.082	1.248–29.636
Fall	2.132	0.699	9.309	0.002	8.429	2.143–33.153
Other	0.520	0.700	0.552	0.458	1.682	0.427–6.630

Cl, confidence interval. *P-values derived from binary logistic regression analysis.

## Discussion

Due to advancements in modern urban infrastructure, more individuals are participating in the construction and transportation industries (8), leading to an increase in car accidents and falls. Reports indicate that nearly 90% of vertebral fractures occur in the thoracolumbar spine, with burst fractures accounting for 30–60% of all thoracolumbar fractures (9–12). TBF is characterized by a fall from height impacting the anterior margin of the vertebral body and injuring the posterior margin (13). Axial loading causes the vertebral body to fracture into multiple fragments, leading to lateral spreading of the pedicles and posterior elements, resulting in a vertical laminar fracture. Laminar fractures have been demonstrated as reliable indicators for assessing the severity of spinal lesions (3). An analysis of patients with TBF and vertebral plate fractures revealed significantly higher injury severity and greater spinal canal encroachment compared to the control group (14). These findings underscore the potential risk association value of laminar fractures in relation to neurologic deficits.

In this study, the ASIA classification was significantly higher in the LF group compared to the NLF group (*P* < 0.05). It is noteworthy that the injury process is dynamic. The presence of neurologic deficits following vertical laminar fractures may result from the impalement of the posteriorly displaced dural sac on the sharp edges of the laminar fracture at the moment of trauma. Additionally, the retraction of the laminar fracture fragments to their original position may lead to neurological deterioration by entrapment of the dural sac and nerve roots when the axial load dissipates (3, 15, 16). The literature has well documented the association between laminar fractures, dural tears, and the severity of spinal lesions (3, 14, 17). Researchers have also sought to identify specific clinical, radiological, or intraoperative factors predicting dural tears and nerve root entrapment (16). Furthermore, scholars have developed diagnostic and treatment methods for cauda equina entrapment in vertical laminar fractures associated with lumbar burst fractures (18). Our study also confirmed that laminar fractures (OR: 0.019, 95% CI: 0.005–0.070), car accidents (OR: 6.082, 95% CI: 1.248–29.636), and falling accidents (OR: 8.429, 95% CI: 2.143–33.153) were independently associated with neurologic deficits after TBF (*P* < 0.05). In both falls and car accidents, the vertebral body undergoes significant longitudinal compression during the injury process, causing the vertebral body to burst into multiple bone fragments. The compression force is transmitted from the vertebral body to the lamina through the pedicle, ultimately resulting in a fissure in the lamina, which leads to neurologic deficits. Additionally, we found that laminar fractures and falling accidents were highly risk association of neurologic deficits (*P* < 0.01). The risk association equation was Logit (P) = −4.358 + 3.535 × laminar fracture – 1.353 × falling accidents.

In the treatment of neurological injuries caused by TBF, internal fixation following decompression is commonly accepted (19). It has been suggested that patients with lumbar burst fractures, particularly those with greenstick laminar fractures or radiological evidence of posterior displacement of neural elements in the injured vertebrae, should undergo posterior surgical exploration (16, 17). Researchers have found that various laminar fracture characteristics impact the surgical outcome of TBF (3). Kyphotic angle (KA) is a critical factor influencing spinal cord injury and neurological deficits following spinal fractures. Studies consistently demonstrate that a greater KA is associated with increased spinal canal narrowing, higher risks of neurological impairment, and post-surgical correction loss (20–22). Particularly, a KA exceeding 20° is often considered a threshold for spinal instability and potential neurological deterioration (21, 22). The presence of posterior element injuries, such as laminar or facet fractures, further exacerbates the risk (20, 21). Imaging assessments, including Cobb angle measurement, play a crucial role in predicting neurological outcomes (22, 23). Early surgical correction of kyphotic deformity is recommended to prevent progressive spinal cord compression and optimize recovery (22). Although spinal canal compromise is widely recognized as a major determinant of neurological deficits and ASIA classification, the role of KA in this relationship remains an important but underexplored aspect. Given the strong association between KA and spinal canal narrowing, KA may serve as an additional predictive measure of neurological outcomes, potentially complementing or even exceeding the predictive value of laminar fractures. However, due to the limitations of the current dataset, a direct comparative analysis between KA, spinal canal compromise, and ASIA classification was not performed. Future studies should incorporate detailed radiological assessments, including spinal canal compromise and KA, to determine their relative contributions to neurological impairment more comprehensively.

However, this study has several limitations. Firstly, it included patients exclusively from a single hospital’s spine surgery department, necessitating a larger, multicenter study to minimize potential bias. Secondly, being retrospective, there is a risk of incomplete clinical data, which could introduce errors, especially given the small sample size. Additionally, potential confounders, such as different spinal fracture mechanisms (e.g., flexion-extension, axial loading), intravertebral MRI findings, and other clinical factors, may have influenced the observed association between laminar fractures and neurological deficits. Since these factors were not comprehensively analyzed in our study, they could contribute to residual confounding effects. Future studies should incorporate a more detailed analysis of fracture mechanisms and imaging characteristics to provide a clearer understanding of their impact on neurological outcomes. In future research, we aim to address these issues by gathering data from multiple centers, conducting prospective investigations, and integrating advanced imaging techniques.

## Conclusion

In this study, we identified laminar fractures, car accidents, and falls as independent risk factors for neurological deficits in patients with TBF. Among them, laminar fractures and falls exhibited high risk association value for neurological impairment. These findings highlight the importance of assessing laminar fractures when evaluating spinal trauma severity and emphasize the need for early intervention in high-risk patients. Furthermore, the risk association model established in this study may provide a reference for clinical decision-making. However, this study has certain limitations. As a retrospective study conducted in a single center, potential selection bias and incomplete clinical data may exist. Future studies should incorporate multicenter prospective designs and advanced imaging techniques to further validate these findings and explore additional factors influencing neurological outcomes.

## Data Availability

The raw data supporting the conclusions of this article will be made available by the authors, without undue reservation.
